# Involvement of Oxidative Stress and Inflammation in Liver Injury Caused by Perfluorooctanoic Acid Exposure in Mice

**DOI:** 10.1155/2014/409837

**Published:** 2014-03-02

**Authors:** Bei Yang, Weiying Zou, Zhenzhen Hu, Fangming Liu, Ling Zhou, Shulong Yang, Haibin Kuang, Lei Wu, Jie Wei, Jinglei Wang, Ting Zou, Dalei Zhang

**Affiliations:** ^1^Department of Physiology, Medical College of Nanchang University, Nanchang 330006, China; ^2^Department of Pathophysiology, Medical College of Nanchang University, Nanchang 330006, China; ^3^Library of Nanchang University, Nanchang 330006, China; ^4^Nanchang Medical School, Nanchang 330006, China

## Abstract

Perfluorooctanoic acid (PFOA) is widely present in the environment and has been reported to induce hepatic toxicity in animals and humans. In this study, mice were orally administered different concentrations of PFOA (2.5, 5, or 10 mg/kg/day). Histological examination showed that the exposure to PFOA for 14 consecutive days led to serious hepatocellular injury and obvious inflammatory cell infiltration. In addition, malondialdehyde formation and hydrogen peroxide generation, indicators of oxidative stress, were significantly induced by PFOA treatment in the liver of mice. Furthermore, hepatic levels of interleukin-6, cyclooxygenase-2, and C-reactive protein, markers of inflammatory response, were markedly increased by exposure to PFOA in mice. These results demonstrated that PFOA-induced hepatic toxicity may be involved in oxidative stress and inflammatory response in mice.

## 1. Introduction

Perfluorooctanoic acid (PFOA), a member of the perfluoroalkyl acid (PFAA) family of compounds, is a strong surfactant that is widely used in the manufacture of lubricants, medical equipment, paper and textile coatings, oil repellents, upholstery, polishes, food packaging, and fire fighting foams [1]. Due to the presence of strong carbon-fluorine bonds, it is practically nonbiodegradable and highly persistent in the environment [2]. PFOA, as well as other PFAAs, has been detected in a variety of environmental matrices from around the globe, including surface waters, air, sludge, soils, sediments, and polar ice caps [2]. Furthermore, detectable levels of PFOA have been found in wildlife and humans [3–5]. In particular, the presence of PFOA and PFOS has been identified in human tissue samples, including liver, kidney, adipose tissue, brain, basal ganglia, hypophysis, thyroid, gonads, pancreas, lung, skeletal muscle, and blood from nonoccupationally exposed subjects [6]. Data from NHANES 1999-2000, 2003-2004, 2005-2006, and 2007-2008 showed that geometric mean PFOA concentrations in serum were 5.2, 3.95, 3.92, and 4.13 ng/mL in the US population, respectively [7].

In recent years, there has been increasing concern regarding potential adverse effects of PFOA on animal and human health. Laboratory studies have shown that PFOA elicits a variety of toxicities, including hepatotoxicity [8], carcinogenicity [9], neurotoxicity [10], mutagenicity [11], developmental toxicity [12], immunotoxicity [13], and genotoxicity [14]. Epidemiologic studies have also demonstrated that PFOA exposure is positively associated with hyperuricemia [15], cardiovascular disease [16], chronic kidney disease [17], thyroid disease [18], and hepatocellular damage [19].

In the body, PFOA is distributed predominantly to the liver and plasma in humans and animals [20]. The liver serves as the main target organ for PFOA, which causes an increased liver weight, hepatocytic hypertrophy, hepatic triglyceride accumulation, multifocal coagulation, and liquefaction necrosis in rodents [8, 21, 22]. In addition, PFOA exposure increases the incidence of malignant hepatocellular carcinoma in rats [23]. Although considerable numbers of studies have reported the adverse effects of PFOA exposure on the liver, the underlying mechanisms have not yet been fully elucidated. Many environmental contaminants have been reported to induce oxidative stress and to result in hepatic injury in experimental animals [24–26]. Moreover, severe environmental pollutants have been implicated to induce hepatic inflammation [27–29]. Therefore, the present study was designed to determine whether PFOA-induced hepatic toxicity was involved in oxidative stress and inflammatory response.

## 2. Materials and Methods

### 2.1. Animals

Male Kunming (KM) mice weighing 20–22 g were purchased from the Laboratory Animal Center of Nanchang University. Mice were maintained at 22 ± 2°C and relative humidity (50% ± 10%) with a 12 h light/dark cycle and acclimatized for 1 week prior to the start of the experiment. All animal procedures were performed in accordance with the Guidelines for Care and Use of Laboratory Animals of Nanchang University and approved by the Animal Ethics Committee of Nanchang University.

### 2.2. Treatments

PFOA (96% purity, Sigma-Aldrich, USA) was dissolved in dimethyl sulfoxide (DMSO). Mice were orally administered different concentrations of PFOA (2.5, 5, or 10 mg/kg/day) once daily for 14 consecutive days. Controls received an equivalent volume of DMSO. At the end of treatment period, the mice were sacrificed after anesthesia with sodium pentobarbital. Blood samples were collected and livers were aseptically excised and weighed. Liver tissues were fixed in 4% paraformaldehyde for histological examination or frozen in liquid nitrogen and then stored at −80°C for biochemical analyses.

### 2.3. Measurement of Serum Enzymes

The blood samples were centrifuged at 13,000 rpm at 4°C for 30 min to separate serum. The activities of serum alanine aminotransferase (ALT), aspartate aminotransferase (AST), alkaline phosphatase (ALP), lactate dehydrogenase (LDH), and total bile acids (TBA) were determined with a biochemical analyzer (7180, HITACHI, Japan).

### 2.4. Histology

The fixed liver samples were dehydrated in ethanol gradient solutions, embedded in paraffin, and sectioned at 5 *μ*m. The sections were stained with hematoxylin and eosin and observed under an optical microscope (IX71 Olympus, Japan).

### 2.5. Measurement of Malondialdehyde (MDA) and Hydrogen Peroxide (H_**2**_O_**2**_)

The levels of MDA and H_2_O_2_ in liver tissue homogenates were measured using commercial kits (Jiancheng Institute of Biotechnology, Nanjing, China), in accordance with the manufacturers' instructions. The analyses were performed with a UV 1800 spectrophotometer (Shimadzu, Japan).

### 2.6. Measurement of Interleukin 6 (IL-6), Cyclooxygenase-2 (COX-2), and C-Reactive Protein (CRP)

The frozen liver tissue was homogenized with ice-cold saline. The levels of IL-6, COX-2, and CRP in liver tissue homogenates were determined using commercially available ELISA kits, in accordance with the manufacturers' instructions (Xitang Biotechnology, Shanghai, China).

### 2.7. Statistical Analysis

Data were presented as the mean ± SEM and evaluated by one-way analysis of variance (ANOVA) and Duncan's multiple-range tests using the GLM procedure of SAS 8.1 software. *P* < 0.05 was considered statistically significant.

## 3. Results

### 3.1. Effect of PFOA on Liver Weight and Morphology

**Figure 1:**
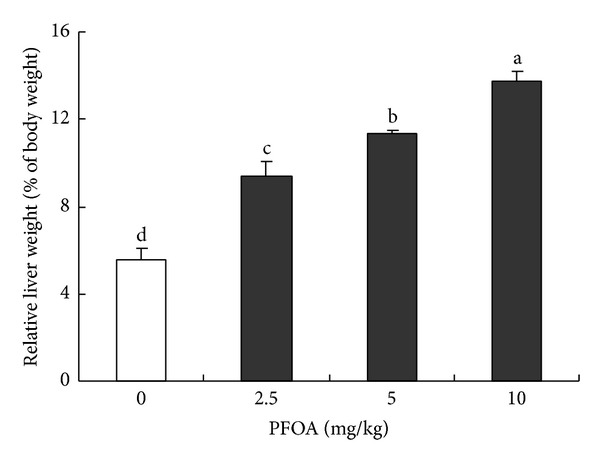
Relative liver weight after exposure to different concentrations of PFOA. Values are expressed as mean ± SEM (*n* = 4). Bars with different letters are statistically different (*P* < 0.05).

**Figure 2:**
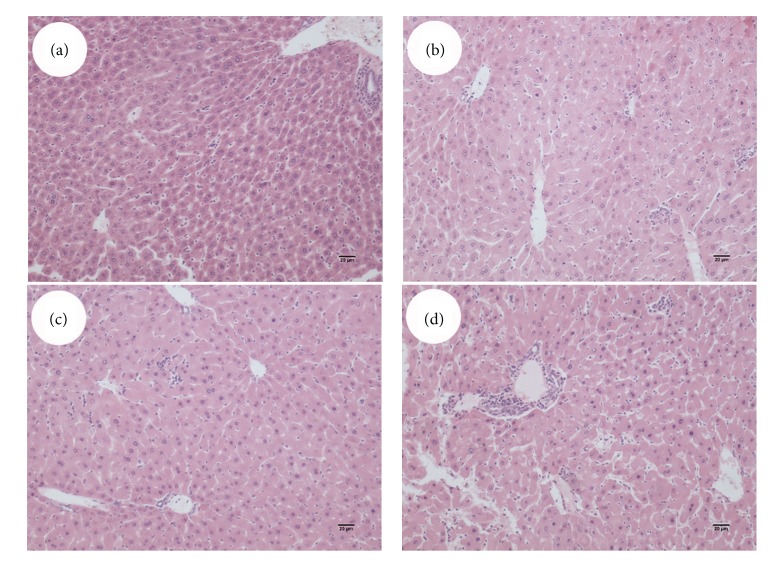
Liver histopathology after exposure to PFOA 0 (a), 2.5 (b), 5 (c), or 10 (d) mg/kg/day for 14 days. Sections of liver were stained with hematoxylin and eosin and then were visualized under an IX71 Olympus microscope. Magnification: 100x.

Oral administration of PFOA (2.5–10 mg/kg/day) for 14 consecutive days caused obvious hepatic hypertrophy and induced a significant increase in the relative liver weight in a dose-dependent manner (*P* < 0.05) (Figure 1). Histological examination of liver sections showed deranged liver architecture, severe edema, vacuolar degeneration, focal necrosis, and obvious infiltration of inflammatory cells in mice exposed to PFOA. The maximal effect was observed at the highest concentration (10 mg/kg/day) (Figure 2(d)) and intermediate effects were found at the doses of 2.5 and 5 mg/kg/day (Figures 2(b) and 2(c)). These adverse histological changes were absent in the liver of control mice (Figure 2(a)).

### 3.2. Effect of PFOA on Serum AST, ALT, ALP, LDH, and TBA Levels

**Figure 3:**
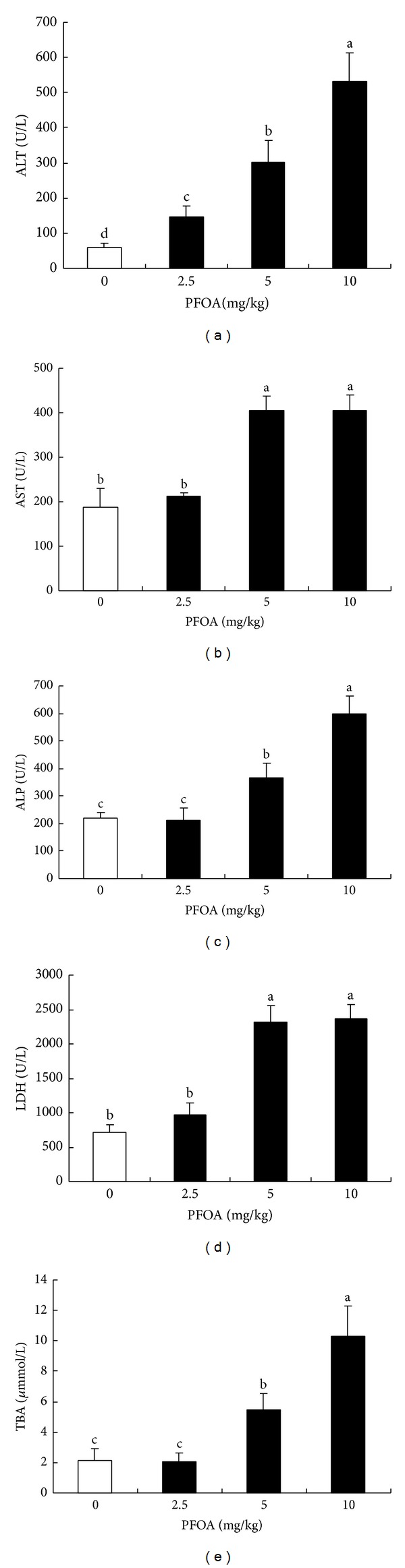
Serum levels of AST (a), ALT (b), ALP (c), LDH (d), and TBA (e) after exposure to different concentrations of PFOA. Values are expressed as mean ± SEM (*n* = 4). Bars with different letters are statistically different (*P* < 0.05).

PFOA administration induced an obvious increase in serum ALT levels in a dose-dependent manner in mice (*P* < 0.05) (Figure 3(a)). Compared with the control, serum AST, ALP, LDH, and TBA levels were significantly increased by treatment with PFOA (5–10 mg/kg/day) (Figures 3(b)–3(e)). There was no significant reduction in these biochemical markers of liver function in the lowest exposure group (2.5 mg/kg/day) compared with the control group (Figure 3).

### 3.3. Effect of PFOA on Liver MDA Formation and H_**2**_O_**2**_ Generation

**Figure 4:**
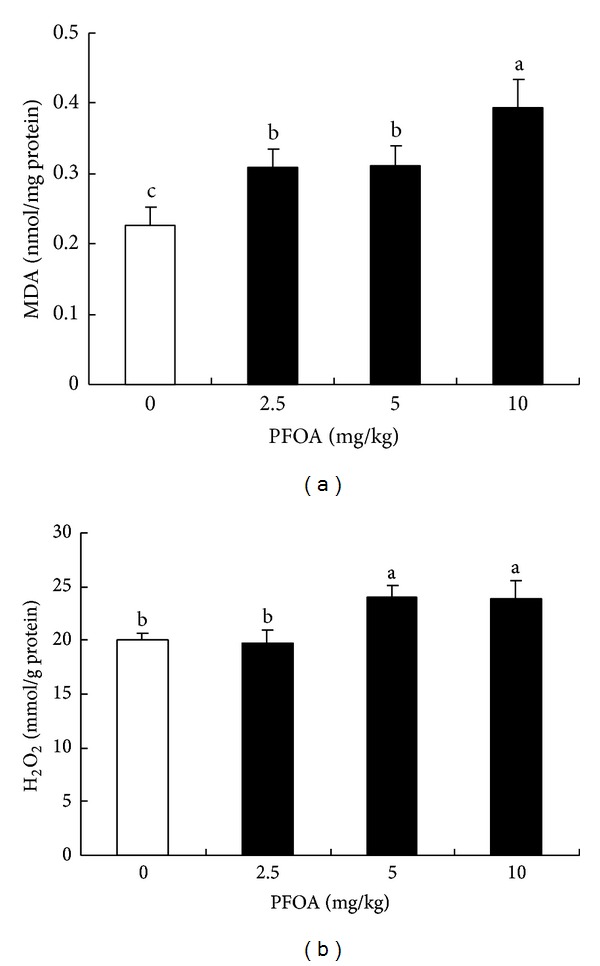
Hepatic levels of MDA (a) and H_2_O_2_ (b) after exposure to different concentrations of PFOA. Values are expressed as mean ± SEM (*n* = 4). Bars with different letters are statistically different (*P* < 0.05).

To explore whether PFOA exposure led to oxidative stress in the mouse liver, two indexes of oxidative stress, MDA and H_2_O_2_, were determined. After PFOA exposure for 14 days, the levels of MDA and H_2_O_2_ in the liver tissue significantly increased compared with the control (*P* < 0.05) (Figures 4(a) and 4(b)). The lowest dose of PFOA had no effect on H_2_O_2_ generation compared with the control (Figure 4(b)).

### 3.4. Effect of PFOA on Liver CRP, IL-6, and COX-2 Levels

**Figure 5:**
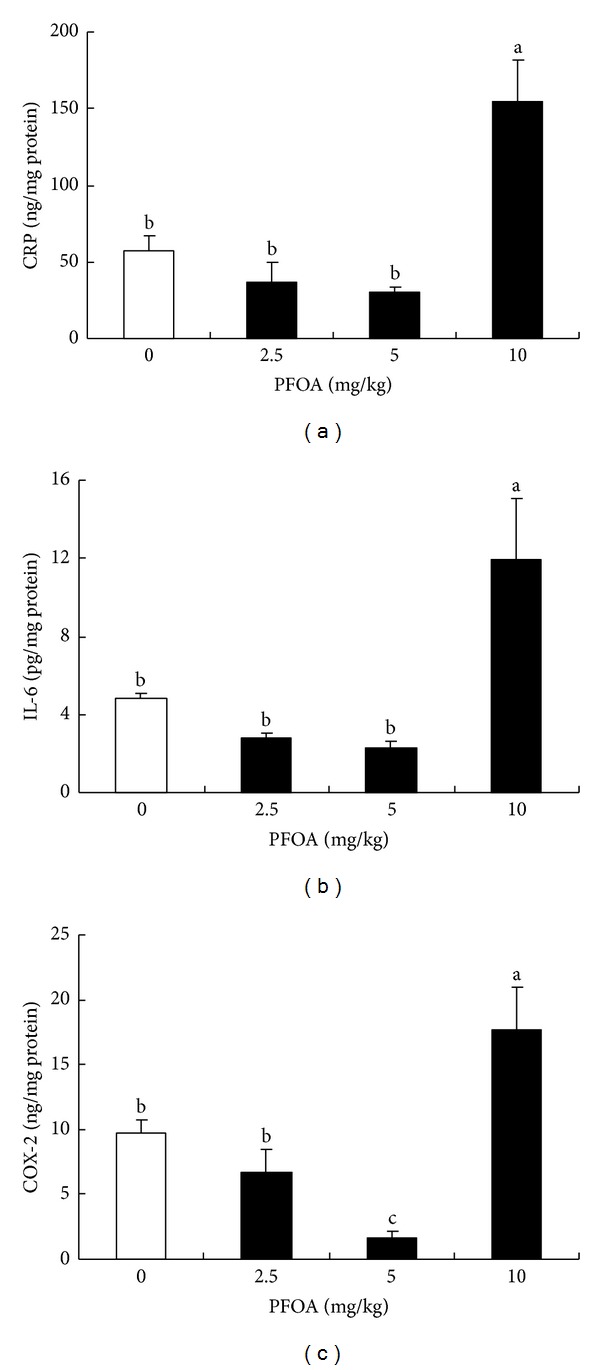
Levels of CRP (a), IL-6 (b), and COX-2 (c) in liver tissue after exposure to different concentrations of PFOA. Values are expressed as mean ± SEM (*n* = 4). Bars with different letters are statistically different (*P* < 0.05).

To investigate whether PFOA exposure-induced liver injury was associated with inflammatory process, three markers of inflammatory response, CRP, IL-6, and COX-2 were detected in liver tissue. After exposure for 14 days, the moderate dose of PFOA (5 mg/kg/day) caused a significant reduction in the hepatic levels of COX-2 compared with the control (*P* < 0.05). However, the high concentration of PFOA (10 mg/kg/day) significantly increased hepatic CRP, IL-6, and COX-2 levels compared with control group (*P* < 0.05). The low-dose exposure to PFOA (2.5 mg/kg/day) did not alter the hepatic levels of the three cytokines (*P* > 0.05) (Figure 5).

## 4. Discussion

Perfluorinated compounds are emerging environmental contaminants of public health concern. Previous studies have shown that PFOA exposure can increase the size and relative weight of the liver in mice [8, 22]. In the present study, oral exposure to PFOA for 14 consecutive days caused obvious hepatomegaly and induced a significant increase in liver weight in a dose-dependent manner. The observation was consistent with the previous studies. In the histopathological evaluation, the liver of PFOA-treated mice showed morphological changes, including structure damage, hepatocellular necrosis, edema, and inflammatory cell infiltration. Moreover, biochemical evaluation indicated that PFOA treatment led to a significant increase in serum enzymes, including AST, ALT, ALP, LDH, and TBA. The leakage of large quantities of serum enzymes into the blood stream was associated with centrilobular necrosis, ballooning degeneration, and cellular infiltration of liver [30]. Previous reports have suggested a positive association between PFOA exposure and serum ALT and AST levels [8, 19]. Our results confirmed the hepatic toxicity of PFOA in mice.

Oxidative stress is considered a critical pathophysiological mechanism in different pathologies, including cardiovascular diseases, cancer, diabetes, rheumatoid arthritis, or neurological disorders [31]. Numerous studies have demonstrated that oxidative stress was an important causative factor in the mechanism of action of environmental contaminants [24–26]. The balance between prooxidant endogenous and exogenous factors and antioxidant defenses in biological systems can be used to assess toxic effects under stressful environmental conditions, especially oxidative damage induced by chemical pollutants [32].

Exposure to PFOA has been demonstrated to generate reactive oxygen species (ROS) and cause oxidative DNA damage in HepG2 cells [14]. However, the increase in ROS production was not concentration-dependent [33]. In cultured tilapia hepatocytes, exposure to PFOA induced a dose-dependent decrease in cell viability accompanied by an increase in MDA formation [34]. In vivo evaluation, PFOA increased the levels of 8-hydroxydeoxyguanosine (8-OHdG), an indicator of oxidative DNA damage, in the liver of* Ppar*α**-null mice but did not elevate 8-OHdG levels in the liver of wild-type mice [35]. In addition, exposure to perfluorononanoic acid (PFNA) and perfluorododecanoic acid (PFDoA) significantly increased the levels of H_2_O_2_ and MDA but inhibited the activities of superoxide dismutase and catalase in the liver of rats [36, 37]. MDA and H_2_O_2_ can be used as indirect measurements of lipid peroxidation and cellular injury. In the present study, PFOA treatment induced an elevation in MDA formation and H_2_O_2_ generation in the liver of mice, suggesting that PFOA-induced hepatic toxicity was related to oxidative stress, which caused lipid peroxidation and hepatocyte injury.

Inflammation is a local immune response to infection and injury. PFOA has been known to induce inflammation by elevating the expression of proinflammatory cytokines tumor necrosis factor *α* and interleukin-1*β* and IL-6 in the spleen and mast cells [38, 39]. In the liver, proinflammatory cytokines produced by hepatocytes participate in hepatotoxic responses [40]. A previous report showed that exposure to PFOA might sensitize hepatic parenchymal cells to other toxicants and thereby aggravate liver injury during acute inflammation [41]. As markers of inflammation, IL-6, CRP, and COX-2 are widely used for estimation of various inflammatory states. In the present study, exposure to a high dose of PFOA (10 mg/kg/day) significantly increased the levels of IL-6, CRP, and COX-2 in the liver tissue of mice. Our results indicated a possible role of PFOA in inflammation and hepatic injury.

## 5. Conclusion

In this study, we showed that oral exposure to PFOA for 14 consecutive days caused an increase in serum AST, ALT, ALP, LDH, and TBA levels and induced hepatocellular necrosis, edema, and inflammatory cell infiltration in mice. In addition, PFOA exposure increased lipid peroxidation and H_2_O_2_ generation and elevated IL-6, CRP, and COX-2 levels in the liver. These results indicated that PFOA could induce hepatotoxicity involving oxidative damage and inflammatory response.
